# Surveillance of *Salmonella* Serovars in the Food Chain in Poland: A Five-Year Review (2016–2020)

**DOI:** 10.3390/pathogens14070712

**Published:** 2025-07-18

**Authors:** Ewelina Skrzypiec, Magdalena Skarżyńska, Magdalena Zając, Renata Kwit, Anna Lalak, Aleksandra Śmiałowska-Węglińska, Emilia Mikos-Wojewoda, Paulina Pasim, Weronika Koza, Dominika Wojdat, Inga Bona, Dominika Pastuszka, Sylwia Hudzik-Pałosz, Dariusz Wasyl

**Affiliations:** Department of Bacteriology and Bacterial Animal Diseases, National Veterinary Research Institute, 24-100 Pulawy, Poland; magdalena.skarzynska@piwet.pulawy.pl (M.S.); rkwit@piwet.pulawy.pl (R.K.); anna.lalak@piwet.pulawy.pl (A.L.); aleksandra.smialowska@piwet.pulawy.pl (A.Ś.-W.); emilia.mikos-wojewoda@piwet.pulawy.pl (E.M.-W.); paulina.pasim@piwet.pulawy.pl (P.P.); weronika.koza@piwet.pulawy.pl (W.K.); dominika.wojdat@piwet.pulawy.pl (D.W.); inga.bona@piwet.pulawy.pl (I.B.); dominika.pastuszka@piwet.pulawy.pl (D.P.); sylwia.hudzik@piwet.pulawy.pl (S.H.-P.); wasyl@piwet.pulawy.pl (D.W.)

**Keywords:** *Salmonella* serovars, salmonellosis, food chain, poultry, livestock

## Abstract

(1) Background: Understanding the distribution of *Salmonella* serovars in food, animals, and their environments is crucial for identifying infection sources and monitoring pathogen prevalence in the food chain. This study analysed *Salmonella* serovars in Poland from 2016 to 2020, focusing on their epidemiological significance. (2) Methods: Isolation of *Salmonella* was carried out following PN-EN ISO 6579 standards, and serotyping was performed using the White–Kauffmann–Le Minor scheme. A total of 7104 isolates were collected from food-producing animals, their environments, food of animal origin, feedingstuffs, and fertilisers. (3) Results: A total of 175 serovars were identified, with *S*. Enteritidis (*n* = 2905; 40.9%), *S*. Infantis (*n* = 1167; 16.4%), and *S*. Typhimurium (*n* = 360; 5.1%) being the most prevalent. Species-specific patterns were observed: *S*. Enteritidis dominated in chickens, ducks, and cattle; *S*. Kentucky in turkeys; *S*. Typhimurium in geese; and monophasic *S*. Typhimurium in pigs. *S*. Enteritidis and *S*. Infantis were most frequent in food of animal origin, especially broiler meat. In feedingstuffs, *S*. Agona was predominant, while fertilisers mostly contained *S*. Derby and *S*. Infantis. (4) Conclusions: The study highlights the source-dependent variety of *Salmonella* serovars and the importance of serotyping in tracing infection routes and preventing the spread of pathogens. Identifying the most common serovars supports the development of targeted preventive measures, including improved biosecurity, hygiene, and management practices to enhance food safety.

## 1. Introduction

*Salmonella* spp. continue to represent one of the principal etiological agents of foodborne illnesses globally, constituting a serious public health concern [1,2,3]. According to estimates by the Centers for Disease Control and Prevention (CDC), the pathogen is responsible each year for approximately 1.35 million infections, 26,500 hospitalizations, and 420 deaths in the United States alone [1]. Data provided by the European Food Safety Authority (EFSA) and the European Centre for Disease Prevention and Control (ECDC) indicate that salmonellosis remains the second most commonly reported zoonosis within the European Union [4,5,6,7,8,9]. During the period 2015–2019, the number of reported cases in EU member states exceeded 90,000 annually, while in Poland it remained between 9000 and 10,000, with over 60% of cases requiring hospitalisation [10]. The highest incidence rates were observed among children aged 0–4 years. In 2020, a marked decrease in the number of reported infections was recorded—52,702 cases in the EU and 5470 in Poland—which has been attributed to the effects of the COVID-19 pandemic resulting in heightened hygiene practices and reduced interpersonal contact [9,11].

In Poland, *Salmonella* Enteritidis has remained the predominant serovar, accounting for 70–92% of cases during the analysed period [10]. Nevertheless, in recent years, the occurrence of other serotypes—such as *S*. Typhimurium (including its monophasic variant), *S*. Infantis, *S*. Derby, and *S*. Kentucky—has been increasingly observed [10,12,13,14,15]. These serotypes are frequently characterised by multidrug resistance and notable adaptive capabilities, posing novel challenges to the efficacy of current preventive measures. The presence of these serotypes has been reported both in humans [10,12,13,14,15] and throughout the food chain [16,17], suggesting potential epidemiological links and possible routes of transmission.

Livestock animals, particularly poultry and swine, serve as the principal reservoirs of *Salmonella*, with eggs and meat constituting typical vectors for the transmission of the pathogen to humans. Despite the implementation of both national and EU-level control programmes, surveillance data continue to reveal persistent contamination across various links in the food production chain—in 2020, 694 foodborne outbreaks caused by *Salmonella* were reported in the EU, nearly 58% of which were attributed to the *S*. Enteritidis, with eggs and pork being the most common sources of infection [9,17]. Moreover, variations in dominant serotypes depending on country, product category, or geographical region—as observed in Belgium, China, Mexico, and Italy [18,19,20,21]—highlight the global dimension of the problem and underscore the necessity for a systemic approach integrating multiple risk factors [22].

Previous national studies described *Salmonella* serovar distribution in Poland for 2005–2015 [16,17] but did not include more recent data or evaluate changes in contamination patterns across sources. This study addresses these gaps by providing the first systematic analysis for 2016–2020, identifying important trends such as the rising prevalence of *S*. Infantis in poultry and food products, increased dominance of the monophasic *S*. Typhimurium variant in swine, and the emergence of *S*. Agona in animal feed.

The objective of the present study is to analyse the distribution of *Salmonella* serovars isolated in Poland between 2016 and 2020 across different stages of the food chain—from livestock populations and animal feed to final food products [17]. Particular emphasis is placed on temporal changes in serovar composition and the identification of potential sources of infection. The findings aim to inform the evaluation of current surveillance and control strategies and to guide future preventive actions in accordance with the “One Health” approach, which integrates the interrelated aspects of human, animal, and environmental health.

## 2. Materials and Methods

In the five-year time period, a total of 11,300 *Salmonella*-suspected isolates were tested in the National Reference Laboratory for Salmonellosis (NRL) at the National Veterinary Research Institute (NVRI). The research material consisted of *Salmonella* strains submitted to NRL by regional veterinary laboratories and those isolated at the NVRI. The isolates were obtained from national *Salmonella* control programmes in poultry, official controls for compliance with feed and food law, research projects conducted at the NVRI, and routine commercial services. The *Salmonella* isolates obtained between 2016 and 2017 were isolated according to the PN-EN ISO 6579:2003 standard [23] and later on according to PN-EN ISO 6579-1:2017 [24]. They were biochemically confirmed to the genus level and a selected set of isolates—comprising 7104 (62.9%) strains—was identified to the serovar level according to the White–Kauffmann–Le Minor scheme [25] using the slide agglutination tests with commercial antisera manufactured by: Biomed, Warsaw, Poland; Immunolab, Gdańsk, Poland; Mast Group Ltd., Bootle, UK; Sifin Diagnostics GmbH, Berlin, Germany; Statens Serum Institut, Copenhagen, Denmark. All isolates that underwent serotyping were included in the analysis; no additional selection was applied. To differentiate *S.* Typhimurium and its monophasic variant 1,4,[5],12:i:- protocol based on the PCR method recommended by the EFSA was used [26,27]. Duplicate strains and isolates without sufficient data on the source of isolation were excluded, defined as repeated isolates with identical serotyping and source metadata from the same sampling point and time.

## 3. Results

### 3.1. Source and Sample Type Distribution

Among the 7104 serotyped *Salmonella* isolates, 66.0% originated from food-producing animals and their environment, followed by food of animal origin (28.0%), feedingstuffs (5.0%), and other sources including fertilisers (1.0%).

Isolates deriving from samples from animals, food, and feedingstuffs were grouped by sample-type categories (Table 1). In the animal group, the majority originated from the livestock environment, while faecal material and internal organs were also frequently represented. In the food category, samples from processing plants and poultry meat (both specified and unspecified) were the most common sources, with *Salmonella* from beef and beef products being rarely detected. In the feedingstuffs category, isolates most often derived from plant-based feed materials, compound feeds for poultry, and a substantial portion came from samples of unspecified or mixed origin.

### 3.2. Serovar Prevalence

**Figure 1:**
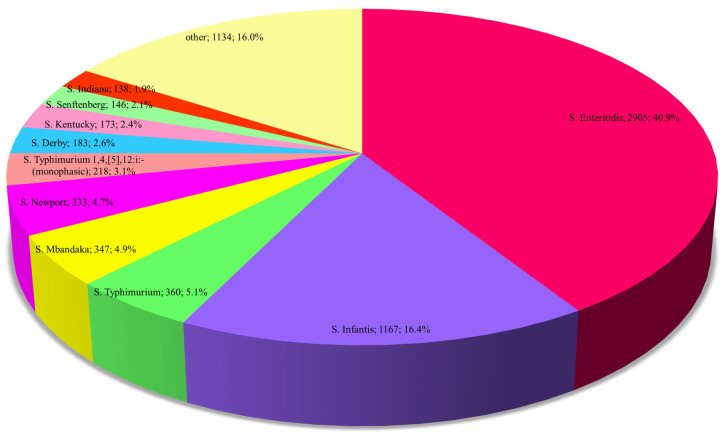
The prevalence of the 10 most frequent serovars identified in the food chain in Poland, 2016–2020. *The pie chart presents both the number of isolates and their percentage share for each serovar*.

A total of 175 *Salmonella* serovars were identified across all isolates. The highest number was observed in animals (*n* = 156), followed by 43 in animal feeds, 42 in food, and 26 in fertilisers. The majority of serovars (*n* = 135) were rarely noted (less than 10 isolates). Overall, *S.* Enteritidis was the most prevalent serovar, accounting for 40.9% of all studied sets. The second most frequent was *S.* Infantis (16.4%). These serovars, together with *S.* Typhimurium, *S.* Mbandaka, and *S.* Newport, accounted for 72.0% of the isolates (Figure 1).

**Figure 2:**
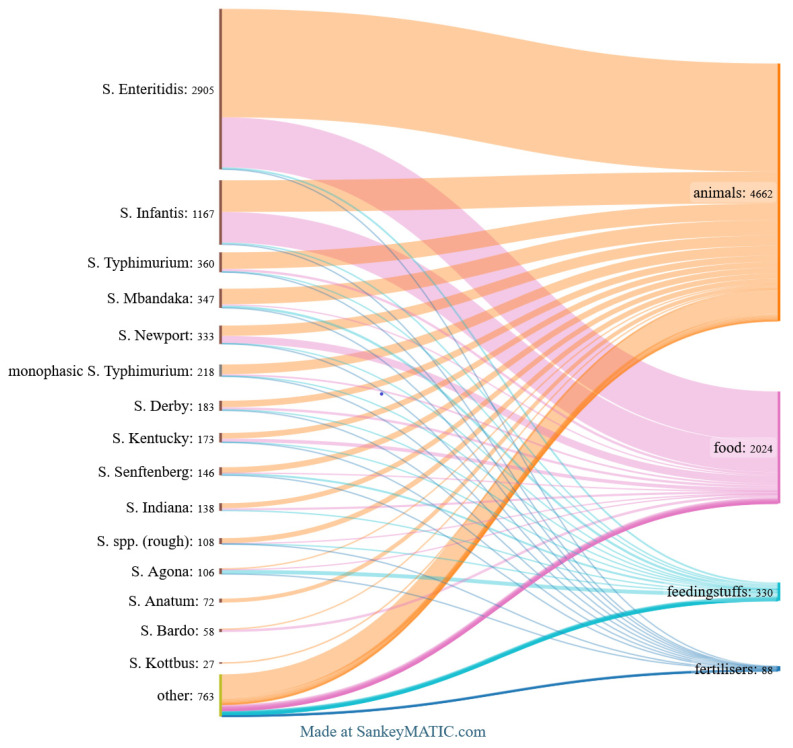
Top *Salmonella* serovars found in animals, food of animal origin, animal feedingstuffs, and fertilisers. *The Sankey diagram illustrates the distribution of the 15 most frequent serovars across these source categories, with flow widths indicating the number of isolates*.

**Figure 3:**
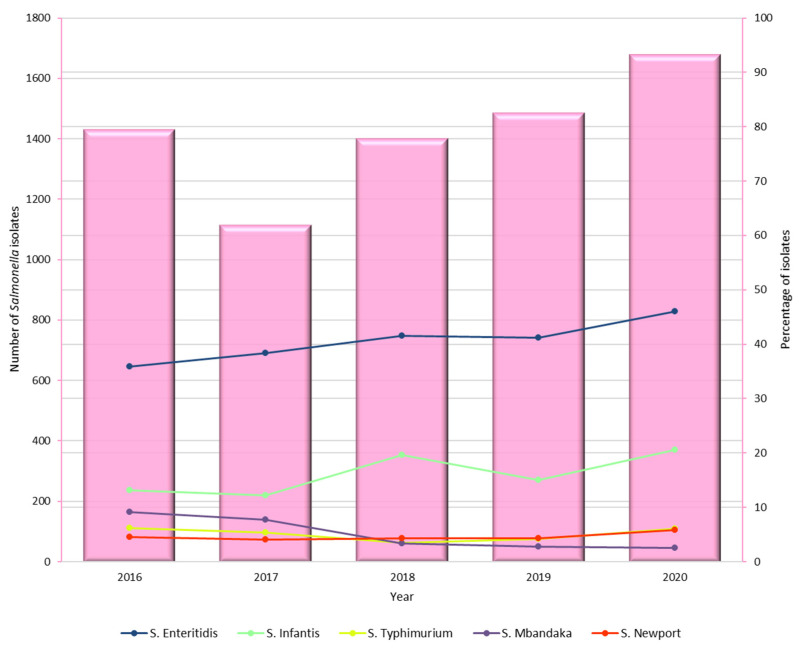
Annual total number of *Salmonella* isolates and percentage of the five most common serovars identified in Poland, 2016–2020. *The combined bar and line chart shows temporal trends in overall isolate counts (bars) and the percentage share of each serovar (lines) over the study period*.

**Figure 4:**
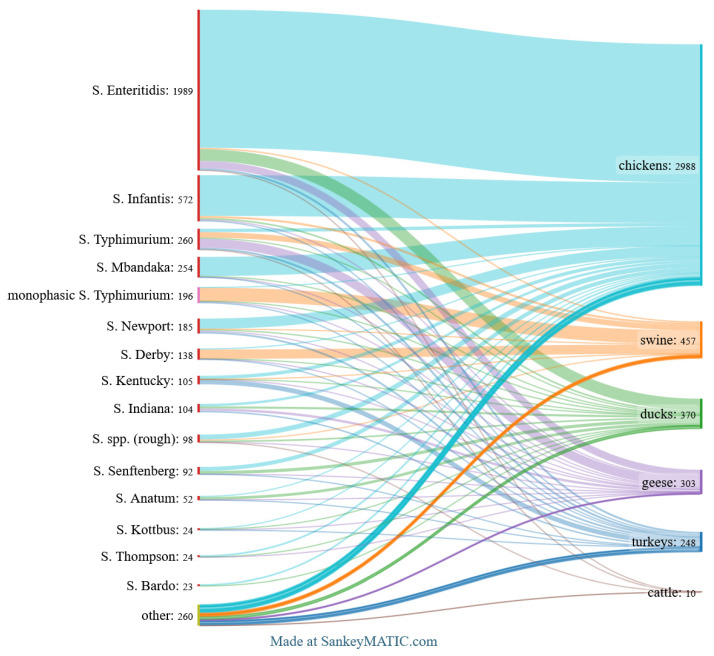
Top *Salmonella* serovars found in animals and their environment. *The Sankey diagram illustrates the distribution of the 15 most frequent serovars by animal species and environmental samples, with flow widths reflecting isolate counts*.

**Figure 5:**
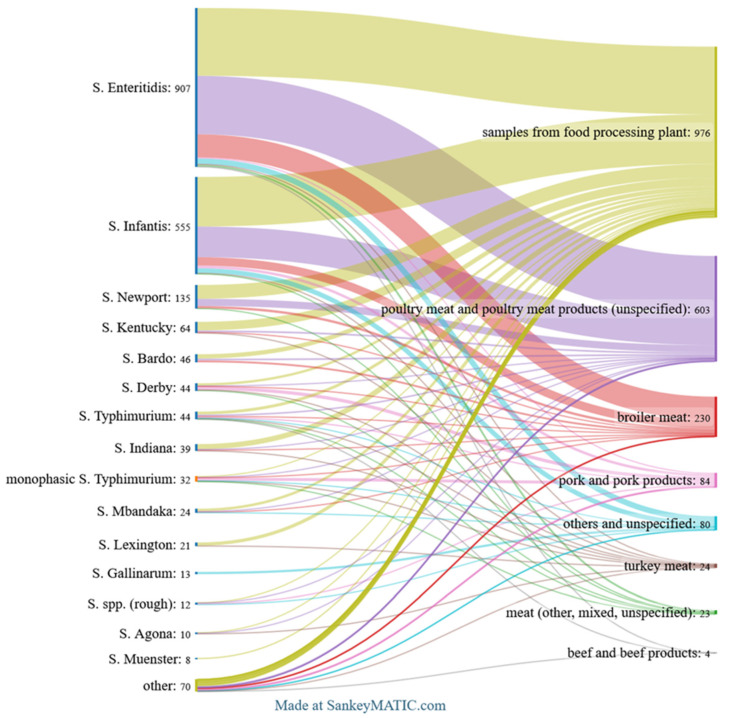
Top Salmonella serovars by food items. *The Sankey diagram displays the distribution of the 15 most frequent serovars across food categories, with flow widths representing the number of isolates*.

The top 15 *Salmonella* serovars identified across all analysed categories are shown in Figure 2. Figure 3, Figure 4 and Figure 5 provide detailed breakdowns for animals and their environment, food of animal origin, and feed materials, respectively.

Out of the 4662 *Salmonella* isolates recovered from animals, the most prevalent serovar was *S*. Enteritidis, accounting for 42.1% of all strains. This was followed by *S*. Infantis (12.3%), *S*. Typhimurium (6.4%), *S*. Mbandaka (6.0%), and *S*. Newport (4.0%). Together, these five serovars represented nearly 71% of all animal isolates.

In the food group, 2024 strains were noted, with the largest number of isolates belonging to *S.* Enteritidis (44.8%), followed by *S.* Infantis (27.4%). Other frequently detected serovars included *S.* Newport (6.7%), *S.* Kentucky (3.2%), and *S.* Bardo (2.3%). Together, these five serovars accounted for the vast majority of food isolates. Less common were *S*. Derby, *S*. Typhimurium, and numerous others, each occurring in small numbers.

Among the isolates from feed, totalling 330 strains, the dominant serovar was *S.* Agona (21.5%), followed by *S.* Mbandaka (12.7%) and *S.* Senftenberg (10.9%). Notable proportions were also observed for *S.* Enteritidis (9.7%) and *S.* Infantis (7.9%). The remaining 38 serovars were detected at lower frequencies, each representing less than 5% of the total.

In the fertilisers group, among 88 isolates, two serotypes predominated: *S.* Derby (17.0%) and *S.* Infantis (14.8%). Other, less numerous but still significant serovars included monophasic *S.* Typhimurium (8.0%), *S.* Newport (6.8%), *S.* Agona, and *S.* Kentucky (each accounting for 5.7%). These overall distributions across source categories are summarised in Figure 2.

The percentage distribution of *Salmonella* serovars identified in Poland between 2016 and 2020 showed notable temporal trends. *S*. Enteritidis consistently had the highest proportion of isolates, increasing from 35.9% in 2016 to 46.0% in 2020. *S*. Infantis also showed a clear upward trend, rising from 13.1% to 20.6% over the same period. In contrast, *S*. Mbandaka decreased steadily from 9.2% in 2016 to 2.5% in 2020. The proportion of *S*. Typhimurium remained relatively stable, ranging from 6.2% to 6.1%, while *S*. Newport showed slight fluctuations but generally increased from 4.5% to 5.8%. Other serovars, including the monophasic variant of *S*. Typhimurium, *S*. Derby, *S*. Kentucky, *S*. Senftenberg, and *S*. Indiana, remained at lower and more variable percentages, typically below 6% each year. For clarity, only the five most prevalent serovars are shown in Figure 3, as including all would result in overlapping lines and reduced readability.

### 3.3. Animal Species-Specific Serovar Distribution

In the animal group, the best-represented isolates were derived from chickens (68.3%). The dominant serovar was *S.* Enteritidis (57.2%), followed by *S.* Infantis (16.9%), *S.* Mbandaka (7.9%), *S.* Newport (4.3%), and rough forms of *Salmonella* spp. (2.0%). Twenty-four serovars appeared fewer than five times, with most appearing only once.

The second-largest group of domestic birds from which strains were isolated was ducks (8.5%). As in chickens, *S.* Enteritidis was the most frequent serovar (39.2%), followed by *S.* Senftenberg (10.5%), *S.* Anatum (9.2%), *S.* Indiana (7.0%), and both *S.* Infantis and rough forms of *Salmonella* spp. (5.4% each).

In geese (6.9%), two serovars predominated: *S.* Typhimurium (36.3%) and *S.* Enteritidis (29.7%). The third most common serovar was *S.* Indiana (9.9%), followed by *S.* Newport (4.3%) and monophasic *S.* Typhimurium (4.0%). Eleven serovars were represented by one or two isolates.

Among turkeys (5.7%), *S.* Kentucky (23.4%) was the most prevalent serovar. Five others occurred in comparable proportions: *S.* Enteritidis and *S.* Typhimurium (each 10.1%), *S.* Newport (9.3%), and *S.* Lexington (7.7%).

In pigs (10.4%), over half of the isolated strains were represented by two serovars: monophasic *S.* Typhimurium (36.8%) and *S.* Derby (25.4%). Less frequently, the following were identified in descending order: *S.* Typhimurium (15.1%), *S.* Infantis (5.7%), and *S.* Enteritidis (3.7%).

Isolates from cattle (0.2%) were scarce, comprising only ten strains and five different serovars, including *S.* Enteritidis, *S.* Typhimurium, *S.* Dublin, *S.* Saintpaul, and the rough form of *Salmonella* spp., with no clearly dominant serovar. These results are shown in Figure 4.

### 3.4. Food and Feed Sources

In the food category, the most frequently detected serovar was *S*. Enteritidis, which appeared in six product groups and accounted for a large share of isolates in food processing plants (39.7%), poultry meat and poultry meat products (55.4%), and broiler meat (57.4%). The second most common serovar was *S*. Infantis, identified in food processing plants (29.0%), poultry meat and products (29.0%), and broiler meat (20.9%). Turkey meat showed a more diverse distribution of serovars, with *S*. Agona and *S*. Infantis being the most frequent (both 16.7%). In pork and pork products, *S*. Derby (22.6%) and monophasic *S*. Typhimurium (20.2%) were the dominant types. Other serovars were found in lower proportions across all food categories (Figure 5).

**Figure 6:**
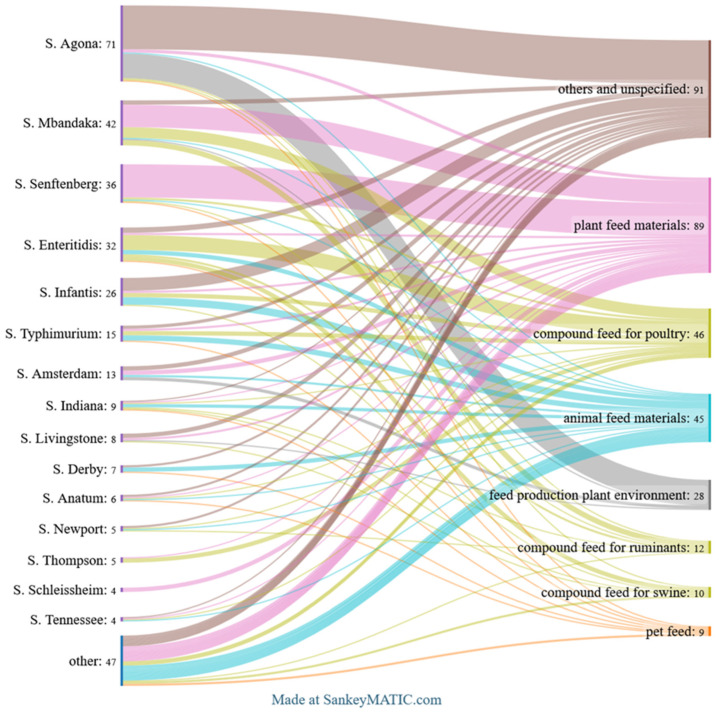
Top *Salmonella* serovars by feed items. *The Sankey diagram shows the distribution of the 15 most frequent serovars across feed categories, with flow widths indicating isolate counts*.

Among feed samples, the distribution of serovars varied by feed type. In plant-based feed materials, the most common serovars were *S*. Senftenberg (34.8%) and *S*. Mbandaka (23.6%). In compound feed for poultry, *S*. Enteritidis (30.4%) was predominant, followed by *S*. Mbandaka (21.7%). In feed for ruminants and swine, *S*. Mbandaka and *S*. Enteritidis were the most frequent, respectively; due to the low number of isolates in these categories, percentages are not shown. In samples from the feed production environment, *S*. Agona was dominant, representing 82.1% of isolates. In animal-derived feed materials, the most frequently found serovars were *S*. Infantis (15.6%) and *S*. Typhimurium (11.1%) (Figure 6).

### 3.5. Statistical Analysis

A chi-square test conducted for the ten most frequently detected *Salmonella* serovars revealed statistically significant differences in their distribution across sample sources (χ^2^ = 922.29; df = 27; *p* < 0.001).

The Simpson’s diversity index (D) quantifies the probability that two randomly selected isolates will belong to different serovars. Lower values of D indicate lower diversity, meaning that a small number of serovars dominate the population. The Simpson’s diversity index revealed notable differences in the variability of *Salmonella* serovars across animals, foods, feeds, and fertilisers. The lowest diversity was observed in chickens (D = 0.635), followed by geese (D = 0.767), pigs (D = 0.773), ducks (D = 0.812), and cattle (D = 0.822). The highest diversity among animal species was found in turkeys (D = 0.899), which was comparable to that observed in animal feeds (D = 0.905) and fertilisers (D = 0.930). The diversity index for foods was D = 0.716, a value similar to that observed in chickens, possibly due to the predominant contribution of broiler meat to this category.

The analysis of 95% confidence intervals (CI) for the most frequent *Salmonella* serovars revealed distinct patterns across sample sources. In animal-derived samples, *S*. Enteritidis accounted for 42.7% of isolates (95% CI: 41.2–44.1%), followed by *S*. Infantis (12.3%, 95% CI: 11.3–13.2%) and *S*. Typhimurium (5.6%, 95% CI: 4.9–6.2%). In food samples, *S*. Enteritidis was again dominant (44.8%, 95% CI: 42.6–47.0%), with *S*. Infantis as the second most common (27.4%, 95% CI: 25.3–29.6%) and *S*. Kentucky ranking third (3.2%, 95% CI: 2.5–4.1%). In feed, the most frequently isolated serovars were *S*. Mbandaka (12.7%, 95% CI: 9.4–16.8%), *S*. Senftenberg (10.9%, 95% CI: 8.0–14.6%), and *S*. Enteritidis (9.7%, 95% CI: 6.9–13.3%). In fertilisers, *S*. Derby was the most prevalent (17.0%, 95% CI: 9.8–27.7%), followed by *S*. Infantis (14.8%, 95% CI: 8.2–24.9%) and *S*. Typhimurium monophasic (8.0%, 95% CI: 3.4–16.0%).

## 4. Discussion

*Salmonella* remains one of the most significant foodborne pathogens, posing a considerable threat to public health worldwide. Systematic monitoring of *Salmonella* serotypes in the food chain is a fundamental component of effective epidemiological surveillance and assessment of national control programmes.

This study presents an updated epidemiological overview of *Salmonella* serotype distribution in Poland for the years 2016–2020, highlighting both the continuation of previous trends and the emergence of new significant shifts. The findings supplement existing data from 2005 to 2015 [16,17,27,28]. There were significant differences in *Salmonella* serovar distribution across sample sources (χ^2^ = 922.29; *p* < 0.001), and diversity index values indicate that serovar composition depends on sample type. Low diversity in chicken and food samples reflects the dominance of *S*. Enteritidis, whereas higher diversity in feed and fertiliser samples (D > 0.9) suggests more heterogeneous contamination. Confidence interval analysis further showed that environmental serovars such as *S*. Mbandaka and *S*. Senftenberg were more common in feed and fertiliser samples. Animal-derived food products remain the main source of human *Salmonella* infections, as supported by the number of isolates identified in this study. *Salmonella*-contaminated feed also poses a substantial risk, potentially introducing novel serotypes into the food chain via livestock production [16,17]. A temporal overview of *Salmonella* serovar distribution across sample sources from 2016 to 2020 reveals both persistent and evolving trends. *S*. Enteritidis remained the dominant serovar in poultry and food samples throughout the study period, indicating a stable yet concerning epidemiological pattern. In contrast, *S*. Infantis exhibited a marked increase in frequency, particularly in food and feed, underscoring its growing significance within the food chain. Meanwhile, the prevalence of classical *S*. Typhimurium declined, while its monophasic variant became increasingly common in swine. Environmental serovars such as *S*. Mbandaka and *S*. Senftenberg were consistently detected in feed and fertiliser samples, although their relative proportions varied between years. These findings underscore the importance of sustained, cross-sectoral surveillance to identify emerging risks and inform integrated control strategies.

As in previous years, *S.* Enteritidis continues to dominate among isolates from chickens, animal-derived food, and geese and ducks, confirming its entrenched presence in the poultry sector. In the analysed period (2016–2020), *Salmonella* Enteritidis maintained its dominant position among isolates from chickens (57.2%), ducks (39.2%), and geese (29.7%). It also constituted a notable proportion of isolates from turkeys (10.1%) and pigs, although its prevalence in swine declined significantly compared to earlier years (19.1% in 2005–2010 [16], with negligible presence in subsequent periods). Compared to previous time frames, a consistently high prevalence of this serovar is evident in the poultry sector, particularly in hens—53.7% in 2005–2010 [16], 54.7% in 2011–2015 [17], and 57.2% in 2016–2020. In food of animal origin, the proportion of *S*. Enteritidis also increased markedly, from 17.6% [17] to 44.8%, which may reflect both its entrenched presence in laying flocks and potential shortcomings in biosecurity or egg quality control systems. Its persistence may also be explained by the ability to colonise reproductive organs and silently contaminate eggs, especially under inadequate on-farm hygiene, rodent control, and hatchery sanitation measures [5,6,9,18]. The continued dominance of *S*. Enteritidis, observed simultaneously in animal populations and food, indicates its high transmission potential and has significant public health implications, particularly in relation to egg-associated outbreaks.

At the same time, a significant phenomenon is the systematic increase in the prevalence of *Salmonella* Infantis, observed across many points of the food chain. This trend is particularly evident in the food sector, where the proportion of this serovar rose from 12.2% to 27.4% [16,17]. A similar increase was observed in poultry isolates, from 12.9% to 16.9%, as well as in swine, where the share rose from 4.5% to 5.7%. This may indicate the ecological and epidemiological success of the serovar [29]. This likely reflects the ecological and epidemiological success of the serovar, driven by antimicrobial resistance, environmental resilience, and the ability to persist in feed and litter. *S*. Infantis was also consistently present in feed throughout all analysed periods, suggesting its potential transmission at various stages of production. A comparable trend has been observed in both Italy and Serbia, where *S. Infantis* dominated among broiler carcass isolates, accounting for 83.3% in Italy [30] and representing the most frequently detected serovar in poultry meat in Serbia [31]. According to data from other EU countries, this serovar is increasingly associated with multidrug resistance and human infections [19,20,29,30,32,33]. It is increasingly associated with multidrug resistance, including ESBL production and resistance to colistin [29,32,34]. These features may facilitate its survival and selection in poultry production systems, as observed in other countries, such as Italy and Serbia [30,31]. These observations highlight the need to include *S*. Infantis in all control programmes, especially considering its growing resistance and widespread presence across the food production environment.

Among poultry, particularly notable changes have been observed in duck and turkey populations. During the 2016–2020 period, several previously less common serotypes, including *S*. Senftenberg, *S*. Anatum, and *S*. Indiana, became more frequently identified. This shift suggests growing serotype diversity and potential changes in sources of infection within this population compared to previous years [16,17]. *S*. Indiana is particularly concerning, as its previously sporadic presence has shown an upward trend. Considering reports of its resistance to quinolones in other countries, this serotype may pose a threat within the national food chain as well [35,36], and the identification of pathogenic, multidrug-resistant *Salmonella* strains in beef from countries outside Europe [37], this serotype may pose a threat within the national food chain as well. Another emerging concern is *S*. Rissen, increasingly detected in Poland and known to be widespread in the United States and parts of Asia, where it constitutes a significant source of human infections [38].

In turkey populations in Poland, significant shifts in *Salmonella* serotype distribution were observed between 2005 and 2020. During 2005–2010, the dominant serotypes were *S*. Saintpaul (32.3%), *S*. Typhimurium (14.5%), and *S*. Newport (9.2%) [16,17]. In the following period (2011–2015), the prevalence of *S*. Kentucky increased to 22.4%, while *S*. Newport rose to 16.0% and *S*. Saintpaul decreased to 13.2% [16,17]. In the most recent period (2016–2020), *S*. Kentucky remained the leading serotype, reaching 23.4%. Additionally, *S*. Enteritidis and *S*. Typhimurium were each recorded at 10.1%. These results indicate a growing serotype variability and a gradual restructuring of dominant types in turkey production. The results indicate that the occurrence of specific serovars is not uniform but closely linked to the environment from which they were isolated, which is important for epidemiological surveillance and the planning of control measures within the food chain. The dominance of a few major serovars alongside many rare ones indicates an uneven distribution. These results demonstrate varying degrees of serovar diversity between sample types, with particularly high heterogeneity observed in feed and fertiliser samples and notably lower diversity in poultry isolates.

Data on swine populations are particularly noteworthy. Since 2010, a clear shift in the dominant *Salmonella* serotype has been observed in pigs, from classical *S*. Typhimurium to its monophasic variant 1,4,[5],12:i:- [26,27,39]. This trend persisted in the years 2016–2020, when the monophasic variant accounted for 36.8% of all porcine isolates, gradually displacing classical *S*. Typhimurium strains, which represented 15.1% in the same period. This trend reflects similar observations in other countries, where monophasic strains—often linked to multidrug resistance and environmental adaptability—are becoming dominant [40]. The rising prevalence of *S.* Derby (from 12.6% in 2005–2010, to 19.6% in 2011–2015, and 25.4% in 2016–2020) may suggest persistence in pork production environments and its zoonotic potential [6,7]. This serotype is frequently reported throughout the swine production chain in countries like Germany, Spain, and Italy, and increasingly in human cases across Europe [9,41].

Feed-related findings also warrant particular attention. *S*. Agona, previously considered sporadic, maintained a prevalence below 10% in 2005–2015 (9.3% and 8.0%, respectively) [16,17], but showed a sharp increase to 21.5% in 2016–2020, which may indicate new contamination routes or the import of contaminated raw materials. Data analysis also shows that *S*. Mbandaka and *S*. Senftenberg are persistent serotypes in the feed production environment, detected across all three periods and reaching 12.7% and 10.9%, respectively, in 2016–2020 [16,17,28]. Similar observations have been reported in Asia and the USA, where feed has been identified as a source of serotypes such as *S*. Mbandaka, *S*. Senftenberg, and *S*. Typhimurium, indicating their environmental persistence and potential to enter the food chain via contaminated feed [42,43]. The prevalence of the latter decreased from 8.2% in 2005–2010 to 4.5% and remained stable thereafter [16,17]. During the same period, *S*. Enteritidis showed a notable increase (from 5.2% to 9.7%), while *S*. Infantis remained consistently present across all timeframes (ranging from 4.9% to 8.9%) [16,17]. The presence of poultry-associated serotypes, such as *S*. Mbandaka and *S*. Senftenberg, in feed suggests their potential transmission through this vector, contributing to infections in production flocks and, indirectly, in humans.

Epidemiological data on the most common sources of *Salmonella* infections in hu-mans during the analysed period are particularly significant. Eggs and egg-based products were the main vectors of *S*. Enteritidis transmission, especially in multinational outbreaks [9]. Poultry meat, including minced and processed products requiring heat treatment, was also a significant source, associated with *S*. Infantis, *S*. Typhimurium, and its monophasic variant [9,19,32]. Pork and pork products constituted the third key source, with *S*. Typhimurium, *S*. Derby, and the monophasic variant being most prevalent [9,41]. Similarly, in the United States, surveillance data indicate that poultry, eggs, and pork products are important sources of human *Salmonella* infections, particularly involving the Enteritidis and Typhimurium serotypes [11,44].

Between 2016 and 2020, the EU reported a stabilisation in salmonellosis incidence, maintaining *Salmonella* as the second most frequently reported zoonosis, after campylobacteriosis. However, despite the implementation of national control programmes, significant localised outbreaks still influenced the overall EU epidemiological landscape. This highlights gaps in surveillance and sanitary control within national food production systems [9,45].

In this context, the “One Health” approach—integrated surveillance of human, animal, and environmental health—is gaining prominence [44,46]. This strategy enables early threat detection, source assessment, and implementation of effective preventive measures throughout the food chain—from feed and farms to the final product reaching consumers. The findings of this study support the practical implementation of the “One Health” approach by highlighting serovar overlaps between animal, feed, food, and fertiliser sources. The detection of the same serovars—such as *S*. Enteritidis, *S*. Infantis, and *S*. Senftenberg—across multiple sectors suggests interconnected transmission pathways within the food chain. For example, the consistent presence of *S*. Mbandaka and *S*. Senftenberg in both feed and animal samples points to possible upstream contamination. These intersectoral links underscore the need for integrated control strategies that address contamination sources early in the production chain, particularly at the feed and environment level, where interventions may have a cascading effect on food safety and public health.

The results of the present study are consistent with data reported at the EU level, where *S*. Enteritidis, *S*. Typhimurium (including the monophasic variant 1,4,[5],12:i:-), and *S*. Infantis remain the most commonly identified serotypes [5,6,7,8,9]. In the context of national data, the dominance of *S*. Enteritidis is evident, as it has remained the prevailing serotype in Poland for many years [16,17]. At the same time, an increasing importance of *S*. Infantis is observed, which may be related to its environmental persistence and frequent resistance to multiple classes of antimicrobials [17]. Particularly noteworthy is the relatively high prevalence of serotypes such as *S*. Mbandaka and *S*. Newport, which, despite having less significance at the EU level, show a distinct regional presence. This may indicate specific local factors, such as farming practices, environmental conditions, or feed sources. It is also worth highlighting the occurrence of poultry-associated serotypes such as *S*. Senftenberg, *S*. Indiana, and *S*. Agona, whose presence, although less common, may be significant in the context of food safety, as well as *S*. Mbandaka [28], which—despite having lower relevance at the EU level—shows persistent presence in the feed and poultry production environment in Poland and holds notable epidemiological importance.

An analysis of time trends shows a continuous and significant increase in the prevalence of *S.* Enteritidis and *S.* Infantis in animal-derived food products [16,17,18,34], confirming their entrenched presence and growing dominance in the food chain. Furthermore, the detection of *S.* Kentucky, *S.* Indiana, and *S.* Newport in food of animal origin [16,17] confirms the wide spectrum of infection risks associated with diverse contamination sources and less typical transmission vectors. The described longitudinal observational study proves that only consistent and integrated actions at all stages of the food chain can ensure lasting reductions in *Salmonella* infections and genuine public health protection.

## 5. Limitations

This study was based on isolates obtained through national control programmes, which may not fully represent all animal species, production stages, or producers outside official surveillance systems. Ready-to-eat (RTE) products, despite their recognised poten-tial as sources of infection, were excluded from sampling. Additionally, other stages of the food chain, such as transport and retail, were not covered, limiting the assessment of serovar occurrence beyond primary production.

Incomplete metadata for a subset of isolates restricted precise source attribution and the analysis of differences in serovar distribution across sectors. Moreover, regional farming practices and environmental conditions may influence the prevalence of specific serovars, potentially limiting the generalisability of these findings to other regions or countries.

These limitations should be carefully considered when interpreting the results and they highlight the need for more comprehensive, harmonised, and integrated surveillance approaches in future research.

## 6. Conclusions

The five-year surveillance data revealed a complex and source-dependent distribution of *Salmonella* serovars in the Polish food chain, with *S*. Enteritidis, *S*. Infantis, and *S*. Typhimurium (including its monophasic variant) predominating in animals, food, feed, and fertilisers. The persistent presence of *S*. Enteritidis in the poultry sector and the in-creasing prevalence of multidrug-resistant *S*. Infantis underscore the need for strengthened preventive and hygiene measures, particularly in poultry and swine production.

The findings confirm the relevance of systematic serotyping as a tool for tracing infection sources and evaluating the effectiveness of control programmes. Expanding surveillance to include antimicrobial resistance profiling and whole genome sequencing would enhance the ability to track transmission routes, detect emerging epidemic-prone serovars, and implement more effective risk management strategies, in line with the “One Health” approach.

## Figures and Tables

**Table 1:** Sample types and number of *Salmonella* isolates from animals, food, and feedingstuffs.

ANIMALS (*n* = 4662)	% (Number of Isolates)
livestock environment (farm)	59.7% (2782)
secretions/excretions (faeces)	27.7% (1290)
internal organs and tissues	7.1% (331)
transportation environment	2.3% (108)
poultry hatching facility	2.2% (105)
swabs/washes/scrapings	0.7% (31)
others and unspecified	0.3% (15)
FOOD (*n* = 2024)	% (Number of Isolates)
samples from food processing plant	48.2% (976)
poultry meat and poultry meat products (unspecified)	29.8% (603)
broiler meat	11.4% (230)
pork and pork products	4.2% (84)
others and unspecified	4.0% (80)
turkey meat	1.2% (24)
meat (other, mixed, unspecified)	1.1% (23)
beef and beef products	0.2% (4)
FEEDINGSTUFFS (*n* = 330)	% (Number of Isolates)
others and unspecified	27.6% (91)
plant feed materials	27.0% (89)
compound feed for poultry	13.9% (46)
animal feed materials	13.6% (45)
feed production plant environment	8.5% (28)
compound feed for ruminants	3.7% (12)
compound feed for swine	3.0% (10)
pet feed	2.7% (9)

## Data Availability

The original contributions presented in this study are included in the article. Further inquiries can be directed to the corresponding authors.
